# Concomitant Talocalcaneal Coalition as a Risk Factor for Early Relapse Following Ponseti Treatment of Idiopathic Clubfoot

**DOI:** 10.3390/diagnostics11091682

**Published:** 2021-09-15

**Authors:** Mudit Shah, Isaac Rhee, Seung Kyu Lee, Mohammed Salman Alhassan, Hyun Woo Kim

**Affiliations:** 1 Division of Pediatric Orthopaedic Surgery, Severance Children’s Hospital, Yonsei University College of Medicine, 50-1 Yonsei-ro, Seodaemun-gu, Seoul 03722, Korea; muditshah160790@gmail.com; 2 Melbourne Medical School, Ground Floor, Medical Building, Cnr Grattan Street & Royal Parade, University of Melbourne, Melbourne, VIC 3010, Australia; isaac.rhee@gmail.com; 3 Department of Orthopaedic Surgery, Severance Hospital, Yonsei University College of Medicine, 50-1 Yonsei-ro, Seodaemun-gu, Seoul 03722, Korea; TERRY1376@yuhs.ac; 4 Department of Orthopaedic Surgery, King Fahad Hospital Hofuf, Al Sulimaniyah 4th, Al Hofuf 36441, Saudi Arabia; hamod_97@hotmail.com

**Keywords:** talocalcaneal coalition, risk factor, early relapse, Ponseti treatment, idiopathic clubfoot

## Abstract

Concomitant talocalcaneal coalition (TCC) in idiopathic clubfeet is not well documented in the literature. The purpose of this study was to describe our experience with very early relapsing idiopathic clubfeet associated with TCC. Although cases have been successfully treated with the Ponseti casting method, all recurred within 2 months of removing the final cast. A single-centre cohort of twelve feet in eight patients treated by a single surgeon between 2006 and 2020 was investigated retrospectively. Recurred cavus with variable degrees of equinus was the earliest findings noted. TCC was incidentally detected during the open reduction of the earliest three feet in our series. Afterwards, ultrasonography was advised as a screening tool for detecting an associated anomaly; however, only the use of magnetic resonance imaging (MRI) was 100% accurate in diagnosing concurrent TCC. All coalitions were cartilaginous and the posterior facet was most commonly involved facet. The average age was 18 months for the coalition resection and open reduction of a dislocated talonavicular joint, and the average duration of follow-up was 52 months. None of the patients showed clinical signs of relapse at the latest follow-up. We recommend that an associated TCC should be considered in very early relapsing idiopathic clubfoot cases.

## 1. Introduction

Idiopathic clubfoot has been traditionally treated using the Ponseti method of serial manipulation and casting, with initial success rates reported between 85 and 90% [1]. However, relapses are relatively common, and a recent systematic analysis found rates >30% [2]. It has been suggested that the recurrence of the deformity is multifactorial in origin. Firstly, this may be due to the underlying pathology of excessive collagen synthesis with progressive retracting fibrosis in the posteromedial compartment of the foot and leg [3]. Secondly, muscle imbalances such as the weakness of foot evertors and hypoplasia of certain muscle groups may be responsible for recurrence [4,5,6]. Therefore, to avoid recurrence, maintaining a well-corrected foot in an abduction orthosis for about 4–5 years of age is recommended; therefore, non-compliance to orthosis is known to be the most important factor for relapse [7,8].

Early relapse is usually encountered within the first 2 years of successfully completing the Ponseti treatment [8,9,10,11,12] and responds relatively well to the repeated application of this method [9,10,13,14]. Late relapsed feet are frequently treated with various types of surgery; however, the Ponseti technique is still recommended as the first treatment irrespective of age or severity of the deformity. Even if a complete correction is not feasible with repeat casting, the severity of the deformity may decrease, and the extent of surgical correction would be minimised [14].

Regarding soft-tissue abnormalities for Ponseti-resistant clubfeet, a recent magnetic resonance imaging (MRI) study showed a wide range of changes, including unique patterns of specific muscle-compartment aplasia/hypoplasia with increased epimysial and intramuscular fat, which were minimally seen in the Ponseti corrected feet [6]. On the contrary, minimal attention has been paid to bony abnormalities; only a few have reported an association between a talocalcaneal coalition (TCC) and rigid equinovarus foot deformity. However, in these reports, the coalitions were either found incidentally during surgical correction of the resistant clubfoot in children of various age groups [15,16,17] or late in adolescence after the foot became severely deformed [18].

Concomitant TCC is not recognized as a possible risk factor for very early relapsing deformity. The purpose of this study was to describe our experience with TCC-associated idiopathic clubfoot and highlight the occurrence of TCC and its prognostic implications. Early diagnosis allowed early definitive treatment in our patients, and we would like to share our results on achieving a plantigrade foot in this study.

## 2. Materials and Methods

### 2.1. Subjects

This study was approved by our hospital’s Institutional Review Board. Electronic medical records and a database containing imaging studies of all patients with congenital clubfoot were examined. From 2001 to 2020, the senior author treated 192 neonates with idiopathic clubfoot using the Ponseti method. Serial manipulation and weekly casting were performed, as described earlier [1]. Tendo-Achilles (TA) tenotomy was performed to correct the equinus when cavus, adductus, and varus deformities were fully corrected, but ankle dorsiflexion remained <10 degrees above neutral [19]. Ultrasonography (USG) was performed to confirm the continuity of TA at the time of final cast removal. We educated the parents to stretch the foot, maintain the correction, and have the patients wear an abduction ankle-foot orthosis.

Eight patients (six males and two females) with twelve idiopathic clubfeet affected by TCC were enrolled in the present study (Table 1). Left and right feet were equally involved in the cohort, and four patients had bilateral involvement. Initially, all the cases were successfully corrected with the Ponseti method, including bilateral feet in one patient referred for rocker-bottom feet deformity that developed after repeated Ponseti treatment for immediately relapsed clubfeet. Concomitant TCCs were later confirmed through open reduction surgery due to the patients’ resistance to the repeated Ponseti technique. All relevant information relating to clinical features and investigations performed was collected, including types of recurring foot deformity, timing and methods of radiologic diagnosis of coalition, and operative findings such as the coalition type and location.

### 2.2. Surgical Technique

**Figure 1:**
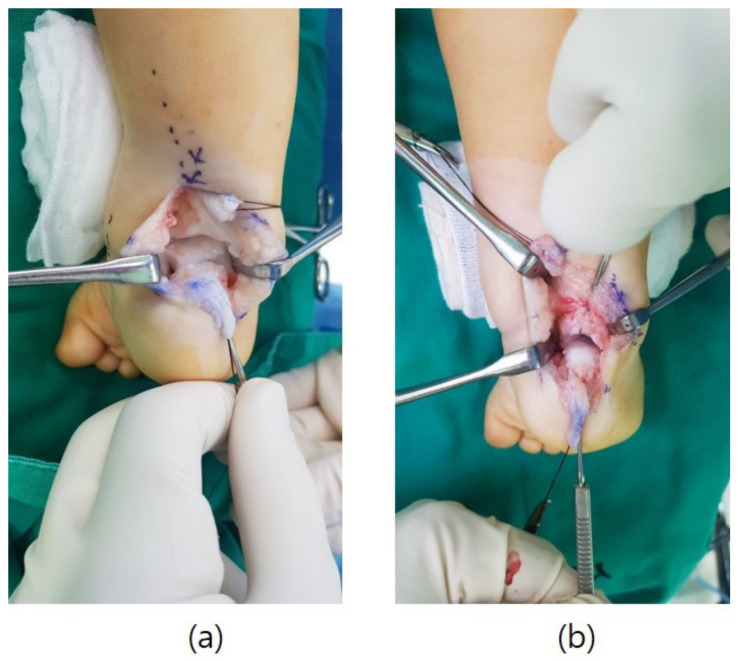
Intraoperative images showing the subtalar joint before (**a**) and after (**b**) resection of a cartilaginous posterior facet coalition from the posterior side of the foot.

After a radiographic diagnosis of TCC, the patients underwent coalition resection, and adequate soft tissue release to reduce the talonavicular joint and achieve a plantigrade foot. The surgical incisions consisted of two separate medial and posterior zigzag incisions [20]. After a Z-lengthening of the TA and retractions of both the peroneal muscles and flexor hallucis longus tendons, the posterior tibiotalar and subtalar joints were sufficiently exposed to visualise and delineate any coalition (Figure 1a). Appreciating the degrees of motion at the ankle and subtalar joints were useful for comparison after performing the coalition excision (Figure 1b).

**Figure 2:**
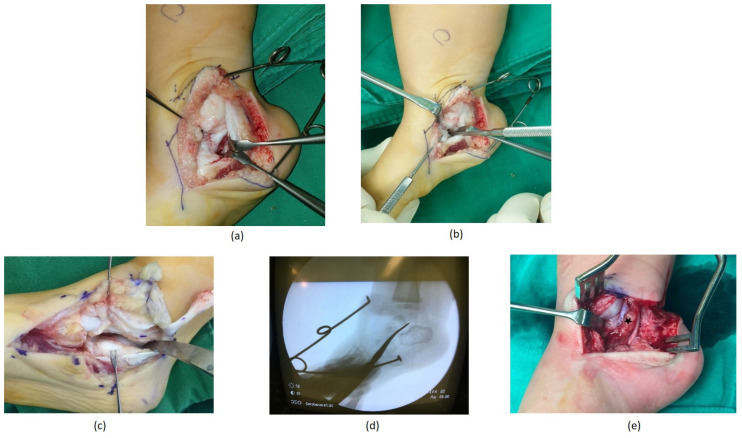
Intraoperative images showing the medial side of the foot. (**a**) Through the medial incision, the flexor digitorum longus tendon retracts with the periosteum to expose the medial aspect of the talocalcaneal joint; (**b**) we attempted to define the extent of the coalition using a blunt instrument; (**c**) after excising the coalition with a scalpel, the entire subtalar joint is visible with normal joint cartilage and the range of subtalar joint motion improves drastically; (**d**) intraoperative fluoroscopic radiographic confirmation of the entire coalition excision; (**e**) to prevent coalition recurrence, a fibrin sealant was used as the interposition material (marked with an asterisk).

A second medial incision was then made to assess any anterior extent of the coalition. The periosteum with the sheath of the flexor digitorum longus tendon was elevated off the bony prominence and reflected volarly (Figure 2a). After incising the periosteum, an anterior and posterior dissection sufficient for identifying the normal joint space was necessary to expose the medial, anterior, and posterior boundaries of the coalition for its complete removal (Figure 2b). The entire coalition was excised using a scalpel. The excision cannot be unnecessarily wide, and the sustentaculum tali must be preserved as much as possible. The coalition was removed until the normal joint cartilage was seen (Figure 2c). At this point, the subtalar joint range of motion should have been markedly improved. We also confirmed the excision of the coalition under the image intensifier fluoroscopy (Figure 2d).

The wound was thoroughly irrigated, and the coalition was cauterised. A fibrin sealant was used as interposition material between the two bony surfaces to prevent any future recurrences of coalition formation (Figure 2e). Any further soft tissue releases were performed as necessary to reduce the talonavicular joint, and a long-leg cast was applied for 6 weeks. At the latest follow-up, the foot was assessed by clinical examination [19] and radiographic measurements of the anteroposterior and lateral talocalcaneal (TC) angles.

## 3. Results

**Figure 3:**
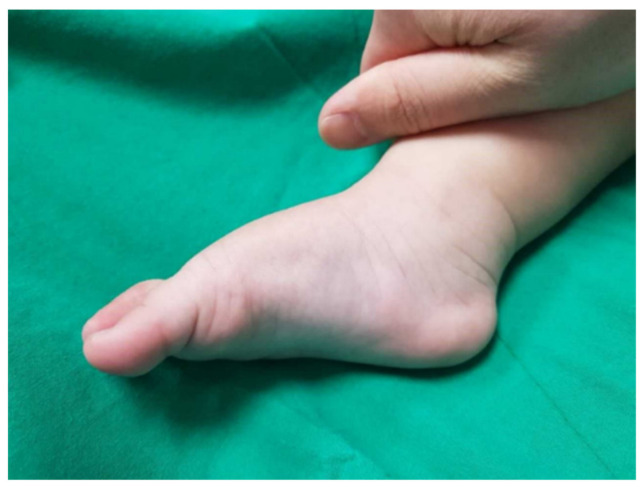
A clinical photograph of a relapsed clubfoot with the plantar flexed 1st ray and mild equinus deformity.

An average of six serial casts were applied, and TA tenotomy was performed in ten feet to correct the primary clubfoot deformity. After removing the final cast, the average relapse time was 7 weeks (range of 3–8 weeks). In all patients, the early presence of cavus with variable degrees of equinus was observed (Figure 3). Forefoot adduction was seen in 42% of feet (five out of twelve), and forefoot pronation was seen in one foot. Repeated Ponseti casting failed to correct the relapsed feet in all patients, and only minimal improvement was noted.

**Figure 4:**
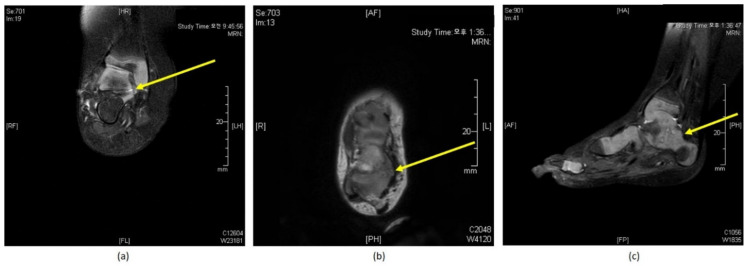
Non-contrast magnetic resonance imaging (MRI) showing talocalcaneal coalition in (**a**) coronal, (**b**) axial, and (**c**) sagittal planes.

In 2006, the senior author oversaw the first case of early-relapsed idiopathic clubfoot associated with TCC; the patient displayed mild degrees of cavus and equinus within one month of removing the final cast, which was resistant to repeat Ponseti treatment. They subsequently investigated the joints and incidentally found a TCC. A resection of the coalition facilitated the reduction of talonavicular and subtalar joints, and subsequent maintenance of a plantigrade foot was possible. After multiple failed attempts at repeat casting for another two of these patients, the senior surgeon performed a USG to check for the continuity of TA and any anomaly of the hindfoot. However, USG was not diagnostic for an associated abnormality in any of the patients. Therefore, for the following nine feet with similar early recurrence, we performed a non-contrast MRI with minimal sequences to confirm the coalition (Figure 4). We were able to accurately diagnose TCC in all nine feet using the MRI. All coalitions were cartilaginous, and one foot had a simultaneous calcaneonavicular coalition.

**Figure 5:**
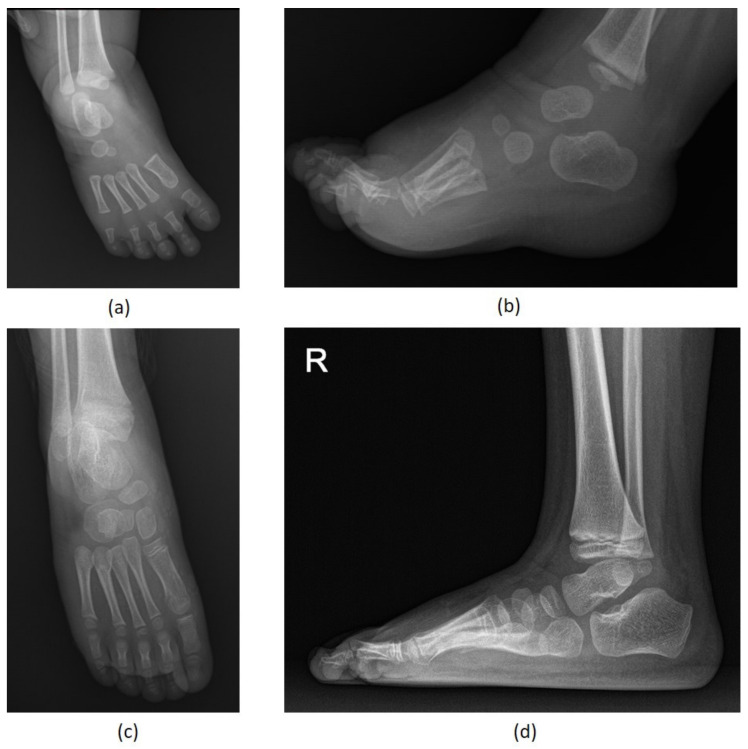
Preoperative anteroposterior (**a**) and lateral (**b**) radiographs of an early relapsed clubfoot with a talocalcaneal coalition that failed to repeat Ponseti casting; anteroposterior (**c**) and lateral (**d**) radiographs at the 38-month follow-up after the coalition resection and open reduction of the talonavicular joint.

The average age for coalition resection was 18 months (range, 4 months–41 months). The most commonly involved facet was the posterior facet (4 out of 12 feet), followed by the involvement of both the middle and posterior facets (3 out of 12 feet). The anterior and middle facets were involved in two feet each, and one foot had all three facet joints. The average duration of follow-up was 52 months (range of 6–173 months). At the latest follow-up, none of the patients experienced any clinical findings suggestive of relapse, and all radiographic measurements were within normal range [21] (Table 2 and Figure 5).

## 4. Discussion

In the first three cases of our cohort, repeat serial manipulation and casting showed only minimal improvement; therefore, the senior surgeon decided to perform a posteromedial release and open reduction of the talonavicular joint. It was during our investigation of the subtalar joints that the coalition was identified and successfully excised. After three such cases, we attempted to establish a protocol to promptly diagnose TCC and allow for the early management of such relapsed clubfeet.

The earliest description of idiopathic clubfoot associated with TCC was in isolated case reports [15,16] and only a few case series thereafter. Spero et al. experienced rigid clubfeet with TCC or calcaneonavicular coalition of congenital and teratologic origins. The coalitions were identified during extensive surgical release or at morbid dissection; however, the authors stated that it was difficult to differentiate a coalition from a malformed joint due to longstanding distorted joints. In addition, only two were radiologically diagnosed before surgery with a plain radiograph and MRI respectively [17]. In Rysselberghe et al.’s study, all patients with idiopathic clubfoot were definitively diagnosed as having coalition only after performing computed tomographic scans when the coalitions had sufficiently ossified; one TCC was diagnosed at age 12 years and four calcaneonavicular coalition after 10 years of age [18].

We are unaware of any studies reporting bony risk factors for early relapse after successful treatment with the Ponseti technique. After the senior surgeon experienced three consecutive clubfeet associated with TCC, we used a non-contrast MRI with minimal sequences and found it to be the diagnostic investigation of choice for clubfoot-associated TCC. To allow earlier treatment of relapsed clubfeet, we attempted to develop a protocol to accurately diagnose the underlying pathology. We believe that surgeons can overlook a coalition if they are not aware of this pathology, and increased awareness of this potential anomaly would help achieve early correction of recurred clubfeet that failed to repeat Ponseti treatment.

The average number of casting in our series was six, comparable to other reported series of Ponseti-treated idiopathic clubfoot [22,23,24]. We believe that the cartilaginous nature of the coalition in neonates allows for minimal resistance to serial manipulation and cast correction. Nevertheless, due to the underlying coalition, the joint may not retain its corrected position with growth; thus, upon removing the final cast, the foot retracts back into the original clubfoot deformity.

Equinus deformity has been reported as the most common clinical finding in early relapses within the first 2 years of successfully completing the Ponseti method [3,10]. However, within 2 months of successful correction in our series of immediate relapses, cavus and variable degrees of equinus were seen in all patients. We do not have a concrete explanation for the relapsed cavus deformity; however, we believe that the aetiology is similar to a primary equinovarus foot deformity, where the first ray of the foot is plantar-flexed, and the forefoot is supinated in relation to the hindfoot. A prospective study with serial radiological assessment or a cadaveric study would be necessary to provide evidence for the development and pathophysiology of this deformity.

As a routine postoperative assessment of the Ponseti treated foot, the senior surgeon began to use USG to examine the continuity of the TA since 2006 [25]. Given the initial experience of incidentally detecting TCC, the senior surgeon advised the radiologists to image the ankle and subtalar joints whilst investigating the continuity of the tendon. Unfortunately, there were no significant findings detected that could rule out an associated anomaly around the joints. We then decided to examine the foot with non-contrast MRI with minimal sequences; the MRI could accurately detect the coalition in the following nine relapsed feet of the cohort. Although USG remained the screening tool of choice for assessing the continuity of the TA in our institute, MRI was the gold standard of investigation to detect and delineate the extent of the TCC in very early relapsing clubfoot. Performing an MRI may not be feasible at all centres due to issues of availability, cost, and anaesthesia; however, we recommend that the surgeon be mindful of the possibility of TCC in immediately relapsing clubfoot presenting with cavus and variable degrees of equinus and utilize MRI with clinical discretion.

Early treatment with a TCC resection and reduction of the dislocated joint has been successful for all our patients. Even after the child began walking, there were no clinical signs of relapse, and the radiographic measurements of anteroposterior and lateral talocalcaneal angles were within normal limits at the latest follow-up. However, our observations have limitations related to the small number of patients, limited follow-up, and inability to predict the long-term results of this study. Furthermore, we suspect that each facet of the subtalar joint in these patients may not become normal even after coalition resection, and the potentially malformed joint orientation may cause another deformity as the child grows.

## 5. Conclusions

We recommend that surgeons maintain a high degree of suspicion for the talocalcaneal coalition in cases of very early relapsing idiopathic clubfoot with cavus and variable degrees of equinus, as this pathology may be more common than previously documented. MRI is the gold standard investigation and should be performed for definitive diagnosis even in the absence of abnormal findings with ultrasonography.

## Figures and Tables

**Table 1:** Details on the patients.

Patient Number	Sex	Affected Foot	Initial Number of Casts Including Final Cast after Tenotomy	Earliest Signs of Relapse	Time to Relapse (in Weeks)	Affected Subtalar Facet	Age at Coalition Resection (in Months)	Follow-Up Duration (in Months)
1	F	Right	5	forefoot pronation, cavus, equinus	3	posterior	4	173
2	M	Right	6	cavus, equinus, forefoot adduction	7	anterior	27	133
		Left	6	cavus, equinus	7	anterior	27	133
3	M	Left	5	cavus, equinus	8	posterior	41	34
4	F	Right	7	cavus, equinus, forefoot adduction	8	middle and posterior	17	38
		Left	7	cavus, equinus, forefoot adduction	8	middle and posterior	17	38
5	M	Left	6	cavus, equinus	7	middle	12	22
6	M	Right	6	cavus, equinus	7	middle	14	14
		Left	6	cavus, equinus	7	middle and posterior	14	14
7	M	Right	6	cavus, equinus, forefoot adduction	8	posterior	16	8
		Left	6	cavus, equinus, forefoot adduction	8	posterior	16	8
8	M	Right	6	cavus, equinus	7	anterior, middle, and posterior	12	6

**Table 2:** Radiographic measurements.

Patient Number	Affected Foot	Preoperative Anteroposterior Talocalcaneal Angle (°)	Preoperative Lateral Talocalcaneal Angle (°)	Anteroposterior Talocalcaneal Angle at Final Follow-Up (°)	Lateral Talocalcaneal Angle at Final Follow-Up (°)
1	Right	20.8	22.2	30.4	37.2
2	Right	24.2	26.9	30.5	26.9
	Left	26.1	27.0	31.4	27.6
3	Left	27.0	1.0	32.9	27.6
4	Right	8.7	17.8	30.8	32.8
	Left	22.1	24.8	31.7	26.8
5	Left	15.9	18.7	30.9	26.5
6	Right	28.8	23.4	31.9	25.8
	Left	25.8	34.1	32.5	25.4
7	Right	20.2	29.3	34.4	33.9
	Left	16.8	38.4	33.4	39.1
8	Right	32.4	30.8	33.9	38.1

## Data Availability

The original contributions presented in the study are included in the article and further inquiries can be directed to the corresponding author.
